# A Novel qPCR Method for Simultaneous Detection and Quantification of Viable Pathogenic and Non-pathogenic *Vibrio parahaemolyticus* (*tlh^+^*, *tdh^+^*, and *ure*R*^+^*)

**DOI:** 10.3389/fmicb.2018.01747

**Published:** 2018-08-02

**Authors:** Ben Niu, Bin Hong, Zhaohuan Zhang, Lili Mu, Pradeep K. Malakar, Haiquan Liu, Yingjie Pan, Yong Zhao

**Affiliations:** ^1^College of Food Science and Technology, Shanghai Ocean University, Shanghai, China; ^2^Laboratory of Quality and Safety Risk Assessment for Aquatic Products on Storage and Preservation, Ministry of Agriculture, Shanghai, China; ^3^Shanghai Engineering Research Center of Aquatic-Product Processing and Preservation, Shanghai, China; ^4^Engineering Research Center of Food Thermal-processing Technology, Shanghai Ocean University, Shanghai, China

**Keywords:** novel qPCR, PMA, pathogenic and non-pathogenic *V. parahaemolyticus*, rapid detection technique, shrimp, clam

## Abstract

Pathogenic and non-pathogenic *Vibrio parahaemolyticus* strains were simultaneously detected and quantified using a novel viable multiplex real-time PCR (novel qPCR). We used a new PCR primer and probe, *ure*R, as a surrogate for detection of the toxin *trh* gene as the primer was better at identifying variant *V. parahaemolyticus trh* strains. The specificity of all primers and probes used in this study were validated on three standard strains of *V. parahaemolyticus*, 42 clinical strains, 12 wild strains, 4 strains of *Vibrio* spp., and 4 strains of other bacteria. Then, propidium monoazide (PMA) was applied to inhibit DNA of dead cell, and the results of PMA optimized treatments were 15 μM concentration, 5 min incubation periods, 15 min light exposure periods and 30 RPM rotational speed, which resulted in time and cost savings. Pathogenic and non-pathogenic strains were quantified using a two-reaction tube method where the *tlh*, *tdh*, and *ure*R genes were amplified. Additionally, standard curves with a 7-log dynamic range were generated for quantifying viable *V. parahaemolyticus* and the amplification efficiencies were 108.68, 105.17, and 115.61% for *tlh^+^*, *tdh^+^*, and *ure*R*^+^*. This novel qPCR accurately monitored *V. parahaemolyticus* contamination rates in shrimps (*Penaeus vannamei*) and clams (*Ruditapes philippinarum*) sampled from retail stores located in a major district in Shanghai. In conclusion, our assay can prioritize the detection and quantification of viable pathogenic *V. parahaemolyticus* and can prove to be a more effective tool for reducing infection risks from consumption of seafood in Shanghai.

## Introduction

*Vibrio parahaemolyticus* is a gram-negative and halophilic pathogen which was identified in the 1950s (Shinoda, 2011). This bacterium is widely distributed in the marine and estuarine environment (Bej et al., 1999) and is a major food borne pathogen in China and South East Asia countries (Su and Liu, 2007; Zhang et al., 2015a). It is the common cause of gastrointestinal disease from consumption of raw or lightly cooked seafood (Letchumanan et al., 2015; Eschbach et al., 2017). The *V. parahaemolyticus* virulence factors contributing to disease include adhesin, thermostable direct hemolysin (TDH), TDH related hemolysin (TRH), 2 type III secretion systems (T3SS1 and T3SS2) and 2 type VI secretion systems (T6SS1 and T6SS) (Ceccarelli et al., 2013; Wang R. et al., 2015). TDH and TRH are considered the major virulence factors: the *tdh* gene, a 567 bp, was cloned and determined by Kaper et al. (1984) and the *trh* gene was determined by Hondo et al. (1987, 1988,1989). There are five variants of the *tdh* gene (*tdh*1, *tdh*2, *tdh*3, *tdh*4, *tdh*5) and these variants share 97% sequence identity (Nishibuchi and Kaper, 1990), while two variants of *trh* gene (*trh*1, *trh*2) only show 84% sequence identity (Nishibuchi et al., 1989; Bisha et al., 2012) and the toxins expressed by *trh*1 and *trh*2 are related to toxins expressed by *tdh* (Ceccarelli et al., 2013; Nilsson and Turner, 2016). The lack of sequence identity of the *trh* gene and the relatedness of the hemolysin toxins can lead to classification errors. A useful surrogate for *trh* is the urease gene cluster *ure*R. The *ure*R gene cluster is immediately upstream of *trh* and using this cluster can circumvent challenges associated with *trh* variation (Suthienkul et al., 1995; Nilsson and Turner, 2016).

Rapid detection technique can improve the management of microbial food safety and lots of detection methods were developed for *V. parahaemolyticus* (Nordin et al., 2017; Wu et al., 2018; Yang et al., 2018). Standard detection for *V. parahaemolyticus*, which is time and labor intensive, consists of isolation, purification and identification based on thiosulfate-citrate-bile-salts-sucrose (TCBS) agar and Polymerase Chain Reaction (PCR) (Niu et al., 2018). This method, however, fails to detect viable but non-culturable (VBNC) cells and *V. parahaemolyticus* has been shown to easily enter VNBC state as a response to adversity (Coutard et al., 2007). The VBNC cell loses the ability to divide but maintains metabolic, gene expression, antibiotic resistance, and pathogenic properties. Nevertheless, this cell can regain its normal culturable state given the right circumstances. Current *V. parahaemolyticus* detection is also unable to discriminate pathogenic from non-pathogenic *V. parahaemolyticus* which can be important from a food safety perspective. PCR assays were developed for the detection of *V. parahaemolyticus* to overcome the shortcomings above and these assays include the real-time qPCR and multiplex qualitative PCR (multi-qPCR) (Xiao et al., 2015; Zhang et al., 2015a). However, qPCR and multi-qPCR generates high false-positive rates due the persistence of DNA after cell death and therefore overestimates infection risks.

There are two major approaches for reducing false-positive rates caused by residual DNA from dead cells. The first approach is real-time reverse-transcriptase qPCR where cDNA is reverse transcribed by RNA (Gonzálezescalona et al., 2009; Miller et al., 2011; Ye et al., 2012). However, RNA extraction is difficult and the rapid degradation of RNA limits its wider application (Bleve et al., 2003; Morin et al., 2004). The other approach is the coupling of qPCR with nucleic acid dyes, ethidium monoazide (EMA) and propidium monoazide (PMA) (Salam et al., 2014; Li et al., 2015; Wang L.J. et al., 2015). In non-viable cells, these nucleic acid dyes selectively penetrate and covalently bind to genomic DNA after photoactivation. PMA is a better dye as EMA is known to also bind to the DNA of viable cells (Kobayashi et al., 2009; Yang et al., 2011; Fittipaldi et al., 2012) and PMA-qPCR is the preferred method for distinguishing viable from non-viable cells (Lee and Levin, 2009; Yang et al., 2011; Fittipaldi et al., 2012).

The *tlh* gene is present in both pathogenic and non-pathogenic *V. parahaemolyticus* strains while the *tdh* and *trh* gene are only present in pathogenic strains. In this study we take into account of the presence of the *tlh*, *tdh*, and *trh* gene by using a two-reaction tube system where one reaction tube only detects the *tlh* gene and the second tube only detects the *tdh* and *trh* genes.

The objective of this study is to establish a novel qPCR method for simultaneous detection and quantification of viable pathogenic (*tlh^+^*, *tdh^+^*, and *ure*R*^+^*) and non-pathogenic (*tlh^+^*, *tdh^-^*, and *ure*R*^-^*) *V. parahaemolyticus* strains. The precision, practicability and robustness of this novel-qPCR method will be compared to the standard method of detection and the novel qPCR method will be used to monitor the *V. parahaemolyticus* contamination rates in retail store samples from a major district in Shanghai.

## Materials and Methods

### Bacteria Strain and Preparation of Inoculum

The 65 bacterial strains used in this study were listed in **Table 1**. *V. parahaemolyticus* strains were grown in 9 mL tryptic soy broth (TSB; Beijing Land Bridge Technology Company Ltd., Beijing, China) with 3% (w/w) sodium chloride (NaCl) and incubated at 37°C for 16–18 h, and other strains were grown in 9 mL TSB at 37°C for 18–20 h to get approximately 9 Log CFU/g.

**Table 1:** Specificity of the novel qPCR amplification of *tlh*, *tdh*, and *ure*R target genes in *V. parahaemolyticus*.

Bacterial Species	Strains	No. of Strains	PCR results		
			*tlh^+^*	*tdh^+^*	*trh^+^*
*V. parahaemolyticus*	ATCC 33847	1	+	+	-
	ATCC 33846	1	+	+	-
	ATCC 17802	1	+	-	+
	V. pc-A	3	+	+	+
	V. pc-B	4	+	-	+
	V. pc-C	34	+	+	-
	V. pc-D	1	+	-	-
*V. vulnificus*	Wild type from shrimp	12	-	-	-
*V. fluvialis*	CGMCC 1.1611	1	-	-	-
*V. anguillarum*	CICC 10475	1	-	-	-
*V. vulnificus*	MCCC 1H00066	1	-	-	-
*V. cholera*	GIM1.449	1	-	-	-
*L. monocytogenes*	ATCC 19112	1	-	-	-
*S. Typhimurium*	CICC 21484	1	-	-	-
*E. coli O157:H7*	ATCC 43889	1	-	-	-
*S. aureus*	CCTCC AB 91093	1	-	-	-

*List of the bacteria information. 1. The gene information of V. pc-A, V. pc-B, V. pc-C, and V. pc-D were certified by Li et al. (2017), see Supplementary Table S1 for more information. 2. ATCC, America Type Culture Collection; USA, CICC, China Center of Industrial Culture Collection; CGMCC, China General Microbiological Culture Collection Center; CCTCC, China Center for Type Culture Collection); MCCC, Marine Culture Collection of China; “+/-” indicated the negative and positive results of PCR, respectively.*

### Preparation of Dead *V. parahaemolyticus*

*V. parahaemolyticus* ATCC 33847 (*tdh^+^*), ATCC 17802 (*trh^+^*), and VP1 (*tlh^+^*) were inoculated in TSB^+^ (TSB with 3% (w/w) NaCl) at 37°C for 12–18 h. Then bacteria suspensions were transferred to 10 mL sterile centrifuge tubes at 3,000 × *g* for 10 min at 25°C, respectively. Afterward, the density of the bacterial suspensions was measured by BioTek Synergy2 (Winooski, VT, United States) at 600 nm (OD_600__nm_). When the OD_600nm_ values of *V. parahaemolyticus* are in the interval (1.2, 1.3), the bacteria concentrations are 9 Log_10_ CFU/g (Niu et al., 2018). Dead cells were obtained by heating suspensions at 85°C for 30 min.

### Optimization of PMA Treatment

Propidium monoazide (PMA) (Biotium, Hayward, CA, United States) stock solution (2 mM) was obtained by dissolving 1 mg PMA in 980°μL ddH_2_O, then stored at -20°C protected from light. All the optimization treatments were carried out with the new machine designed by Zhang et al. (unpublished). To optimize the treatment of PMA, both dead and viable cells (approximately 8 Log_10_ CFU/g) were subjected to PMA treatment where five concentrations of PMA (10, 15, 25, 50, or 100°μM), five incubation periods (5, 10, 15, 20, or 30 min), and seven light exposure periods (5, 10, 15, 20, 25, 30, or 40 min), and six rotation rates (10, 20, 30, 40,50, or 60 RPM) were tested.

### DNA Extraction

Bacterial DNA was extracted by using the TIANamp Bacteria DNA Kit (Tiangen Biotech Beijing Co., Ltd., Beijing, China) according to the manufacturer’s instruction, whereas modification was made: DNA was eluted from adsorption column twice by adding 100 μL (one-time 50 μL).

### qPCR and Standard Curves

All primers and probes were synthesized by Invitrogen Corp (Shanghai, China) unless otherwise stated. TaqMan primers and probes used in this study for detection and quantification of *V. parahaemolyticus* were listed in **Table 2**. The novel method was carried out in a final volume of 20:2 μL of 10 × PCR Buffer (Invitrogen, United States), 1.2 μL of 50 mM Mg^+^ (Invitrogen, United States), 0.5 μL of 10 mM dNTPs mix (Invitrogen, United States), 0.5 μL of 10 μM primer, 0.2 μL of Taq DNA polymerase (5 U/μL) (Invitrogen, United States), and 0.2 μL of 10 μM probe were used for each strain; 1 μL of DNA were used as template. A Quant Studio^TM^ 6 Flex Real-Time PCR system (Applied Biosystems, Foster City, CA, United States) was used with the following thermal profile: initial denaturation of genomic DNA at 95°C for 1 min, followed by 40 cycles of denaturation at 95°C for 5 s, and annealing at 59°C for 45 s.

**Table 2:** TaqMan primers and probe sequences used in this study.

Primer/probe	Sequence, 5′–3′	Target	References
*tlh*-F	ACTCAACACAAGAAGAGATCGACAA	*tlh*	Nordstrom et al., 2007
*tlh*-R	GATGAGCGGTTGATGTCCAA	*tlh*	
*tlh*-P	VIC/CGCTCGCGTTCACGAAACCGT/BHQ1	*tlh*	
*tdh*-F	TCCCTTTTCCTGCCCCC	*tdh*	Nordstrom et al., 2007
*tdh*-R	CGCTGCCATTGTATAGTCTTTATC	*tdh*	
*tdh*-P	NED/TGACATCCTACATGACTGTG/MGB	*tdh*	
*ure*R-F^a^	GCGTAGTCATCGTTCCGAAATAC	*trh*	Nilsson and Turner, 2016
*ure*R-R^a^	AAGTGAGCCTCCATTGATTGTAGAG	*trh*	
*ure*R-P^a^	FAM/TCGCGTATC/ZEN/CTGCACTCTAACACCCA/3IABKFQ/	*trh*	

*^*a*^Synthesized by Integrated DNA Technologies, Coralville, IA.*

Even for the same target bacteria, some studies have shown that the PCR effect would be still influenced by different target genes or different target sequence lengths (Chang et al., 2010; Soejima et al., 2011; Li and Chen, 2013). Additionally, Anand Løvdal found that the optimization of PMA would also be affected by different proportion of dead and viable bacteria (Pan and Breidt, 2007; Løvdal et al., 2011). Therefore, three target genes *tlh*, *tdh*, *ure*R were optimized in different proportion of dead and viable bacteria to obtain the optimization treatments: (1) viable cell (approximately 2–7 Log_10_ CFU/g) without PMA treatment; (2) Mixed bacteria solution (for example: 1 mL 9 Log_10_ CFU/g dead cell and 1 mL 8 Log_10_ CFU/g viable cell were added into 8 mL TSB to get the mixed bacteria solution (8 Log_10_ CFU/g dead cell and 7 Log_10_ CFU/g), then diluted to get the required mixed bacteria solution): viable cell (approximately 2–7 Log_10_ CFU/g) and dead cell (approximately 3–8 Log_10_ CFU/g) without PMA treatment; (3) Mixed bacteria solution: viable cell (approximately 2–7 Log_10_ CFU/g) and dead cell (approximately 3–8 Log_10_ CFU/g) with PMA treatment; (4) Mixed bacteria solution: viable cell (approximately 2–7 Log_10_ CFU/g) and dead cell (approximately 1–6 Log_10_ CFU/g) with PMA treatment. Then the optimized proportion of dead and viable bacteria was set for the standard curve.

### Validation of the Novel qPCR

In order to evaluate the robustness of this assay, *V. parahaemolyticus* VP1 (*tlh^+^*), ATCC 33847 (*tdh^+^*), and ATCC 17802 (*trh^+^*) were employed to assess the target gene *tlh*, *tdh*, and *ure*R, respectively. As for *V. parahaemolyticus* VP1 (*tlh^+^*), approximately 9 Log_10_ CFU/g was obtained by measured the OD_600_ value in the interval [1.2, 1.3]. Afterward, it was diluted and inoculated into the 9 mL TSB^+^ medium to achieve the cell concentration with 2–8 Log_10_ CFU/mL, and then plated the bacteria solution on TCBS agar at 37°C, 48 h, in the meantime, 1 mL bacteria solution were transferred to1.5 mL centrifuge tube for qPCR. Both ATCC 33847 (*tdh^+^*), and ATCC 17802 (*trh^+^*) were enumerated and validated in this way.

### Detection of Practical Samples

Shrimp (*Penaeus vannamei*) and clam (*Ruditapes philippinarum*) were purchased from various regions of Shanghai, including Baoshan District, Jiading District, Main urban District, Qingpu District, Songjiang District, Minhang District, Jinshan District, Fengxian District, and Pudong New Area (this Area is divided into the northern region and southern region, Nanhui New Area). The water samples of shrimp (*Penaeus vannamei*) and clam (*Ruditapes philippinarum*) were collected and put into empty Nongfu Spring bottle at the same time. All the samples were stored in the mobile refrigerator and transported to the laboratory in time, processed in 24 h, and then stored at -80°C.

The whole shrimp and the whole clam tissue were placed in the biological safety cabinet and weighed by scales. Twenty five were added into 225 mL 0.1% aseptic Peptone Water (PW). Then they were homogenized in a stomacher (Bag Mixer 400VW, Interscience, France) for 2 min and 2 mL homogenate were transferred into 5 mL centrifuge tube for 1 min (200 × *g*, 4°C), 1 mL supernatant transferred into 1.5 mL centrifuge tube (2 min, 12,000 × *g*, 4°C) for DNA extraction (according to the manipulation above). Water sample were filtered from nitrocellulose filter membrane (Millipore, Billerica, MA, United States) by the vacuum pump (Millipore, Billerica, MA, United States), afterward, the bacteria were washed by ddH_2_O from filter membrane for PMA treatments prior to DNA extraction (Fast DNA Spin Kit for Soil, Millipore, Billerica, MA, United States). And all the DNA were stored at -80°C. All experiments in this study were repeated three times independently.

## Results

### Specificity of Primers and Probes

The specificity of this method was tested by 65 strains (**Table 1**), of which there were three standard strains of *V. parahaemolyticus*, 42 strains of clinical *V. parahaemolyticus* (see Supplementary Table S1), 12 strains of wild *V. parahaemolyticus*, 4 strains of *Vibrio* spp., and four strains other bacteria. As shown in **Table 1**, all the tested *V. parahaemolyticus* were positive for *tlh.* The *tdh* and *trh* independent strains only showed positive signals for *tdh* and *ure*R fluorescent probe, respectively. All non-target strains were negative for *tlh*, *tdh*, and *ure*R fluorescent probe, which indicated the high specificity of this method.

### PMA Optimization

**FIGURE 1:**
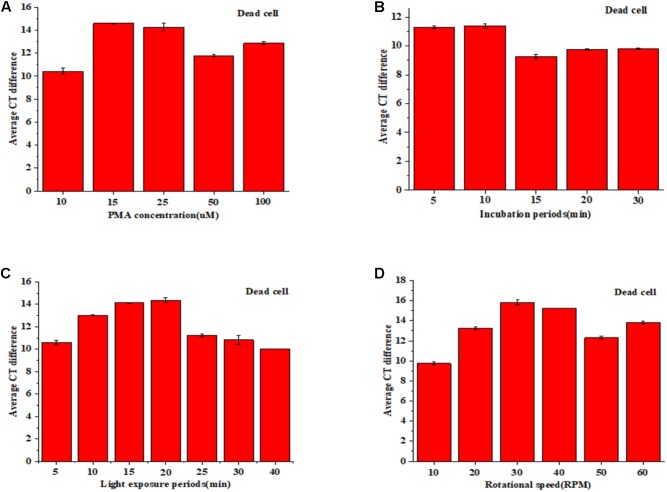
PMA pretreatment on dead cell: concentration **(A)**, incubation time **(B)**, light exposure **(C)**, and rotation rate **(D)**.

As shown in **Figure 1A**, the horizontal coordinate was PMA concentration with 10, 15, 25, 50, and 100 μM, respectively. And vertical coordinate was the average difference value of Ct that is result of Ct value of the viable cell without PMA treatment minus the Ct value of the same concentration dead cell with PMA treatment. There was no significant difference (*p* < 0.05) between 15 and 25 μM of PMA concentration. While the concentration was more than 25 μM, the effect even became worse. **Figure 1B** is a schematic effect on incubation. There was no significant difference (*p* < 0.05) between 5 and 10 min of incubation time, so 5 min was selected as the incubation treatment. For light exposure, the treatment effect did not decrease until 20 min (*p* < 0.05) (**Figure 1C**), so the shorter time of 15 min was selected as the light exposure. In addition, the rotation on treatment effect was addressed for the first time. **Figure 1D** is a diagram of rotation treatment, and 30 RPM was chosen as best rotation rate.

Moreover, in order to avoid the possible inhibition of PMA on viable cell, viable cell experiments on the same conditions were conducted. The result was shown in Supplementary Figure S1. All data indicate that the optimization conditions were 15 μM PMA concentration, 5 min incubation time,15 min light exposure, and 30 RPM rotation rate.

### Standard Curve

**FIGURE 2:**
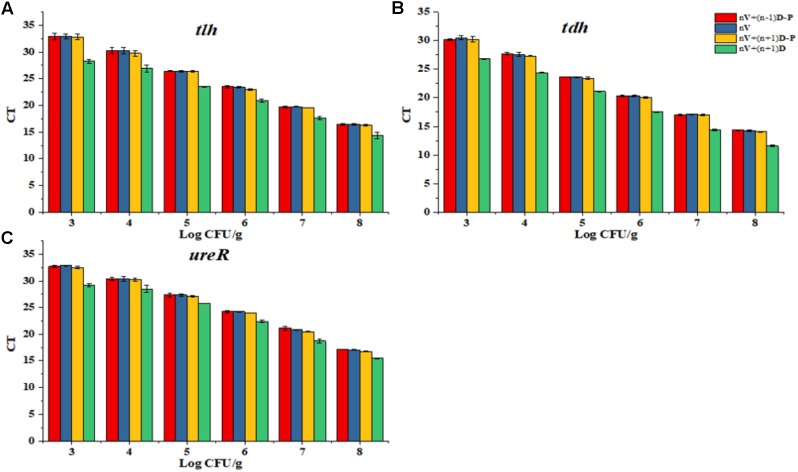
Determination of the proportion of dead and viable cell for the standard curve. **(A)** Proportion of dead and viable cell for *tlh* primer. **(B)** Proportion of dead and viable cell for *tdh* primer. **(C)** Proportion of dead and viable cell for *ure*R primer.

As shown in **Figure 2**, *tlh*, *tdh*, and *ure*R were treated with different concentration ratio of dead and viable cell, respectively. Results showed that the PMA modification effect was most close to the DNA amplification of viable cell, when the concentration of dead bacteria was less than viable cell by 1 Ct. So, we chose this concentration ratio of viable and dead cell for future standard curve.

**FIGURE 3:**
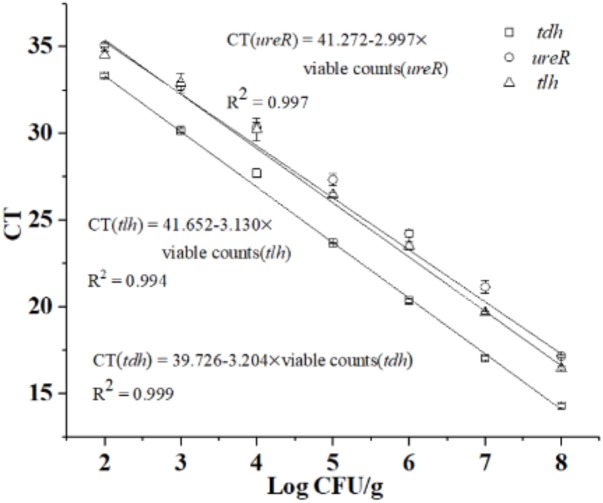
Standard curves of *tlh*, *tdh*, and *ure*R in novel qPCR.

Based on PMA optimization and concentration ratio of dead and viable cell above, the constructed standard curves of *tlh*, *tdh*, and *ure*R were shown in **Figure 3**. Results showed that *R*^2^-value was 0.994, 0.999, and 0.997 for *tlh*, *tdh*, and *ure*R, and the slopes of the three standard curves were -3.130, -3.204, and -2.997, respectively. The amplification efficiency (*E* = 10^-1/slope^-1) of the corresponding PCR calculated in this method were 108.68, 105.17, and 115.61%, respectively. The results indicated that the novel qPCR reaction system had good amplification efficiency and was suitable for further analysis.

### Validation of the Novel qPCR

After the validation of specificity, limit of detection (LOD) and amplification efficiency of the method, we compared this novel method with the standard method. In order to evaluate the efficiency of the novel qPCR’s detection and quantification capability of viable pathogenic and non-pathogenic *V. parahaemolyticus*, a series of 10-fold bacteria dilutions (10^2^–10^8^ CFU/g) were enumerated by both novel and the standard method. As shown in **Table 3**, the result of *tlh* counted from TCSB was agreed the PCR results with approximately 10^2^–10^8^ CFU/g (*p* < 0.05). Thus, comparing to traditional plate counting method, the results demonstrated that our novel qPCR provided a robust and efficient means for the detection and quantification of non-pathogenic *V. parahaemolyticus*. The same conclusions could easily be drawn for both *tdh* and *ure*R from **Table 3**.

**Table 3:** Comparison between novel PCR and traditional coating counting method.

Different genes	Inoculated concentration (CFU/g)	Plate counting results (log CFU/g)	PCR results (log CFU/g)
*tlh^+^*	10^8^	8.54 ± 0.45	8.31 ± 0.09
	10^7^	7.38 ± 0.18	7.28 ± 0.00
	10^6^	6.43 ± 0.42	6.57 ± 0.03
	10^5^	5.19 ± 0.61	5.39 ± 0.01
	10^4^	4.14 ± 0.32	4.26 ± 0.01
	10^3^	3.24 ± 0.25	3.27 ± 0.09
	10^2^	2.04 ± 0.43	2.23 ± 0.12
*tdh^+^*	10^8^	8.45 ± 0.33	8.16 ± 0.03
	10^7^	7.12 ± 0.51	7.00 ± 0.01
	10^6^	6.33 ± 0.61	6.29 ± 0.05
	10^5^	5.26 ± 0.42	5.49 ± 0.02
	10^4^	4.36 ± 0.53	4.30 ± 0.05
	10^3^	3.27 ± 0.24	3.53 ± 0.06
	10^2^	2.02 ± 0.12	2.37 ± 0.06
*ure*R*^+^*	10^8^	8.51 ± 0.29	8.71 ± 0.09
	10^7^	7.41 ± 0.53	7.67 ± 0.01
	10^6^	6.17 ± 0.24	6.56 ± 0.04
	10^5^	5.10 ± 0.21	5.24 ± 0.02
	10^4^	4.27 ± 0.22	4.17 ± 0.04
	10^3^	3.17 ± 0.34	2.96 ± 0.04
	10^2^	1.92 ± 0.47	2.20 ± 0.07

### Practical Samples Detection

In order to ensure the detection accuracy of the practical sample, PCR inhibition control in all the samples were conducted (Supplementary Figure S2). And vertical coordinate was average Ct difference that is result of Ct value of the viable cell with PMA treatment minus the Ct value of the same concentration dead cell with PMA treatment. The results of experiments indicated that using of shrimp and clam tissue matrix spiked with purified DNA did not affect the sensitivity of detection, which were about one Ct value more than those obtained with the blank control. Also, water sample spiked with purified DNA had little influence with <1 Ct value. This suggested that the presence of sample homogenate either had no inhibition or had a too small inhibition on the sensitivity of detection.

**FIGURE 4:**
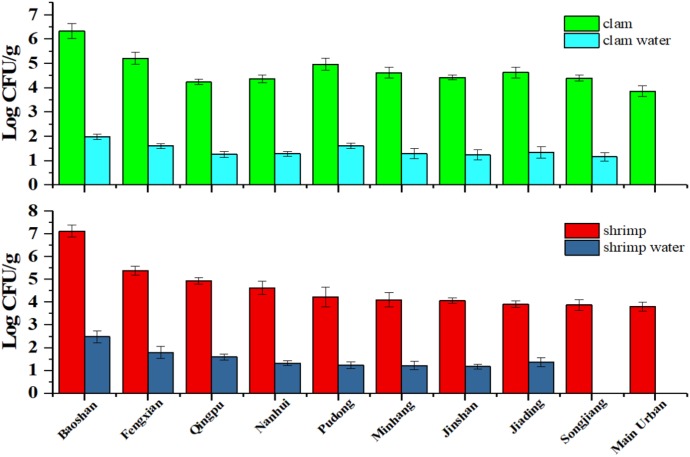
The *V. parahaemolyticus* detection results of shrimp and clam and its water sample collected from retail stores in Shanghai.

As shown in **Figure 4**, we could disclose that *V. parahaemolyticus* contamination rate in shrimp in Baoshan District was as high as 7 log, and the contamination rate in clam was as high as 6 log, which was the highest detection rate in all District. This indicated that these products might pose a risk to consumer when processed with no adequate cooking or eaten in unprocessed state (Zhang et al., 2017).

On the contrary, the contamination rate of *V. parahaemolyticus* detected in Main urban District was lower than other districts, and the viable bacteria were not found in the water samples (it may be lower than LOD). Moreover, contamination rate of *V. parahaemolyticus* in the corresponding water sample is in accordance with the contamination rate of the sample. Interestingly, no-pathogenic *V. parahaemolyticus* was found in all regions, which is consistent with previous reports that the aquatic products in market do not burden a great infection risk (Zhang et al., 2015a).

## Discussion

Microbiological detection and quantification technique had aroused our interest, especially when some sporadic epidemics or pandemic disease caused by pathogens. As shown in Supplementary Table S2 and **Table 4**, time-consuming, low sensitivity, complicated operation, and labor-consuming impeded the progress of traditional methods, on the contrary, PCR based method endowed with better sensitivity, higher throughput and specificity had been popular among researchers. To circumvent the variation of *trh* strains, *ure*R was used in this study. Additionally, innovative means by using two reaction tubes were put forward to overcome the complex logical relationship between *tlh*, *tdh*, and *trh*. Viable qPCR is a typically essential requirement especially when we need to detect and investigate the pathogens which are just present in low amount.

**Table 4:** Comparison between novel PCR and other method.

References	Pretreatment			Reaction time	Discrimination on pathogenicity of V. P	Remarks
	Dye style	Reagent dosage	Treatment time		
Bej et al., 1999	/	/	/	1 h 33 min	*tlh*, *tdh*, *trh*	It is a comprehensive detection of *V. parahaemolyticus* in selfish.
Garrido et al., 2012	/	/	/	55.5 min	*tdh*, *trh*1, *trh*2	It is a detection only for pathogenic *V. parahaemolyticus*.
Robert-Pillot et al., 2010	/	/	/	/	R72H, *tdh*, *trh*	Different reaction conditions were conducted on three target gene.
Eschbach et al., 2017	/	/	/	1 h 2 min	*tlh*, *tdh*, *trh*1, *trh*2	*V. parahaemolyticus*, *V. cholerae* and *V. vulnifcus* were only conducted in interlaboratory study.
Takahashi et al., 2005	/	/	/	1 h 8 min	*tox*R, *tdh*, *trh*	It has been employed in pure culture, seawater, and shellfish.
Zhang et al., 2015b	/	/	/	52 min	*tlh*	*V. parahaemolyticus*, *L. monocytogenes*, and *Salmonella* spp.
Panicker et al., 2004	/	/	/	1 h3 min	*tlh*, *tdh*, *trh*, ORF8	*V. vulnificus*, *V. parahaemolyticus*, and *V. cholerae*.
Lee and Levin, 2009	EMA	2.381 μM	15 min	1 h 35 min	/	*V. vulnificus*.
Zhang et al., 2015a	PMA	100 μM	30 min	52 min	*tlh*	*V. parahaemolyticus* and *L. monocytogenes*
This study	PMA	15 μM	20 min	34 min	*tlh*, *tdh*, *ure*R	The *ure*R was based on Nilsson and Turner (2016).

When it comes to the quantitative technique for viable cell, the difficulties of RNA extraction, and the rapid degradation of RNA hindereNA hindered its application (Bleve et al., 2003; Morin et al., 2004). As for nucleic acid dyes, EMA, the inability to selectively detect viable cells was the most important obstacle impeding the application (Kobayashi et al., 2009; Yang et al., 2011; Fittipaldi et al., 2012). PMA combined with qPCR had been constructed for simultaneous detection and quantification of viable *V. parahaemolyticus*. The impact of rotation rate on PMA treatment was mentioned for the first time in this study, and the usage of PMA was reduced to 15 μM, which is much smaller than the previous report (Zhang et al., 2015a) and results in low detection costs. We have shortened the PMA optimization treatment by 20 min, which was lower than many other studies (Xiao et al., 2015; Zhang et al., 2015a). It is very important for the government to make a quick respond and give a crucial decision-making in a short time especially after the sudden outbreak of disease caused by pathogens.

As shown in **Table 3**, there is a little discrepancy between the novel qPCR and traditional standard method, even though some studies already indicated the consistencies between them (Xiao et al., 2015; Zhang et al., 2015a). Apart from the experimental error, some scenarios still should be taken into account, because the results of PCR are higher than the plate counting. The cells in VBNC state and presence of sublethal cells should be addressed this query. The VBNC cells cannot be cultured in selective medium, however, they could be amplified by PCR. In addition, although some sublethal cells lose the ability to colony and growth, they still have metabolic activity, gene expression, and pathogenicity with membrane integrity (Lleó et al., 1998; Del Mar Lleo et al., 2007). Higher viable cell enumeration by PCR was observed in the first scenario results, whereas the second generates higher enumeration on plate counting. The incubation time in recovery medium might explain this phenomenon. Before coating on agar, comparing to longer time in recovery medium, a short incubation could reduce the discrepancy (Andorrà et al., 2010; Shi et al., 2011). Long enough incubation times allowed the sublethal cell recover in favorable condition, and the viable cell might divide.

There was a discrepancy between seafood sample and its corresponding water sample when the PCR inhibition experiment was conducted. Compared with water sample, seafood sample had a higher inhibition effect on PCR, which might be caused by the complexity culture matrix (Wagner et al., 2008; Varma et al., 2009). Interestingly, the clam sample had a great inhibition effect than shrimp, which was similar to previous finding reported by Umeda and Yoshinaga (2012). We speculated that more mineral such as various metal ions might be enriched by clam, which has a stronger effect on PCR reaction.

During the process of practical sample detection, pathogenic *V. parahaemolyticus* was not found in any samples, which indicated that the seafood in Shanghai market has little infection risk for people (Zhang et al., 2017). Of all sample collected in Shanghai, Baoshan had a highest *V. parahaemolyticus* contamination level, while *V. parahaemolyticus* contamination level in Main urban District was lower than other districts. The low contamination level in Main urban District might be caused by more strict regulations and supervisions. It was clearly seen that there were a lower detection results in the water sample when the counterpart seafood sample had a lower *V. parahaemolyticus* contamination level. Thus, we suggested that the detection of water sample could be reflect the contamination level of practical sample.

Being an important tool, microbiological rapid detection technique is indispensable for the government department after the outbreak of disease and also reliable for disease prevention and control. Furthermore, it is also very necessary for company to make policy decision of production, especially, the Hazard Analysis and Critical Control Point (HACCP) and Quantitative microbiological risk (QMRA) (Notermans et al., 1994; WHO Food Safety Programme, 2002). Finally, the method has been successfully applied to the detection of practical samples, providing a scientific tool for public health and safety.

## Author Contributions

YZ, YP, and HL conceived and supervised the study. BN, BH, and ZZ designed the experiments. BN, BH, and LM performed the experiments. BN, BH, LM, and ZZ analyzed the data. BN, ZZ, PM, and YZ revised the paper. BN wrote the paper.

## Conflict of Interest Statement

The authors declare that the research was conducted in the absence of any commercial or financial relationships that could be construed as a potential conflict of interest.
