# Supplementary material for: A Novel qPCR Method for Simultaneous Detection and Quantification of Viable Pathogenic and Non-pathogenic Vibrio parahaemolyticus (tlh+, tdh+, and ureR+)
Source: Front Microbiol. 2018 Aug 2;9:1747. doi: 10.3389/fmicb.2018.01747 (PMC6083054; doi:10.3389/fmicb.2018.01747)
Supplement: Supplementary file 1 [file Data_Sheet_1.PDF]

Supporting Information

A novel qPCR method for simultaneous detection and quantification of viable pathogenic and non-pathogenic *Vibrio parahaemolyticus* (*tlh*$^+$, *tdh*$^+$, *ure*R$^+$)

Ben Niu,[1†] Bin Hong,[1†] Zhaohuan Zhang[1†], Lili Mu[a], Haiquan Liu[1,2,3,4]

Yingjie Pan[1,2,3], YongZhao[1,2,3*]

[1] College of Food Science and Technology, Shanghai Ocean University, 999# Hu Cheng Huan Road, Shanghai, 201306, China

[2] Laboratory of Quality & Safety Risk Assessment for Aquatic Products on Storage and Preservation (Shanghai), Ministry of Agriculture, 999# Hu Cheng Huan Road, Shanghai, 201306, China

[3] Shanghai Engineering Research Center of Aquatic-Product Processing & Preservation, 999# Hu Cheng Huan Road, Shanghai, 201306, China

[4] Engineering Research Center of Food Thermal-processing Technology, Shanghai Ocean University, Shanghai 201306, China

Table S1 The gene information (*tlh*, *tdh*, and *trh*) of clinical *Vibrio parahaemolyticus*.

Strains Name	*tlh*$^{+}$	*tdh*$^{+}$	*trh*$^{+}$
V. pc1	+	+	-
V. pc2	+	+	-
V. pc15	+	+	-
V. pc16	+	+	-
V. pc17	+	+	-
V. pc18	+	-	+
V. pc19	+	+	-
V. pc20	+	+	-
V. pc21	+	+	-
V. pc22	+	+	-
V. pc25	+	+	-
V. pc26	+	+	-
V. pc27	+	+	-
V. pc28	+	+	-
V. pc29	+	+	-
V. pc32	+	+	-
V. pc33	+	+	-
V. pc34	+	+	-
V. pc35	+	+	-
V. pc36	+	-	+
V. pc37	+	+	-
V. pc38	+	+	-
V. pc40	+	+	-
V. pc41	+	+	-
V. pc42	+	+	-
V. pc43	+	+	+
V. pc44	+	-	-
V. pc45	+	+	-
V. pc46	+	+	-
V. pc47	+	+	-
V. pc48	+	+	-
V. pc49	+	+	+
V. pc50	+	+	-
V. pc51	+	+	-
V. pc54	+	+	+
V. pc55	+	+	-
V. pc85	+	-	+
V. pc89	+	+	-
V. pc90	+	+	-
V. pc94	+	-	+
V. pc97	+	+	-
V. pc100	+	+	-

Table S2 Comparison of qPCR, RNA-qPCR and Plate counting assays with the developed novel qPCR

Name	mechanism	Time	Labor	Accuracy	DOL	Throughput	Cost	Remarks
Plate counting	Colony-Forming Units	4-5 day	High	Low	10 CFU/100mL	Low	Low	False-negative results in case of VBNC cell.
qPCR	DNA amplification	~4 h	Moderate	Moderate	10^2 cell/mL	High	High	No discrimination between dead and viable cells.
RNA-qPCR	DNA amplification	>4 h	Moderate	Moderate	10^2 cell/mL	High	High	Difficulty on RNA extraction
novel-qPCR	DNA amplification	~4 h	Moderate	High	10^2 cell/mL	High	High	No

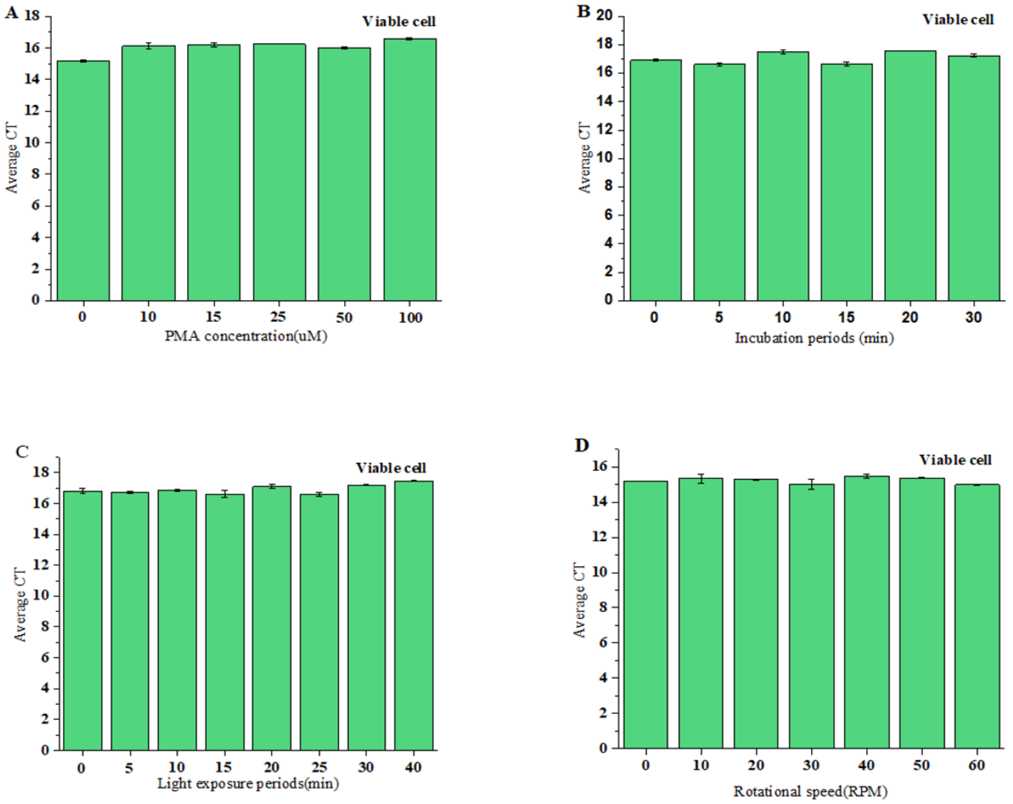

Fig. S1 PMA pretreatment on viable cell: concentration (A), incubation time (B), light exposure (C), and rotation rate (D).

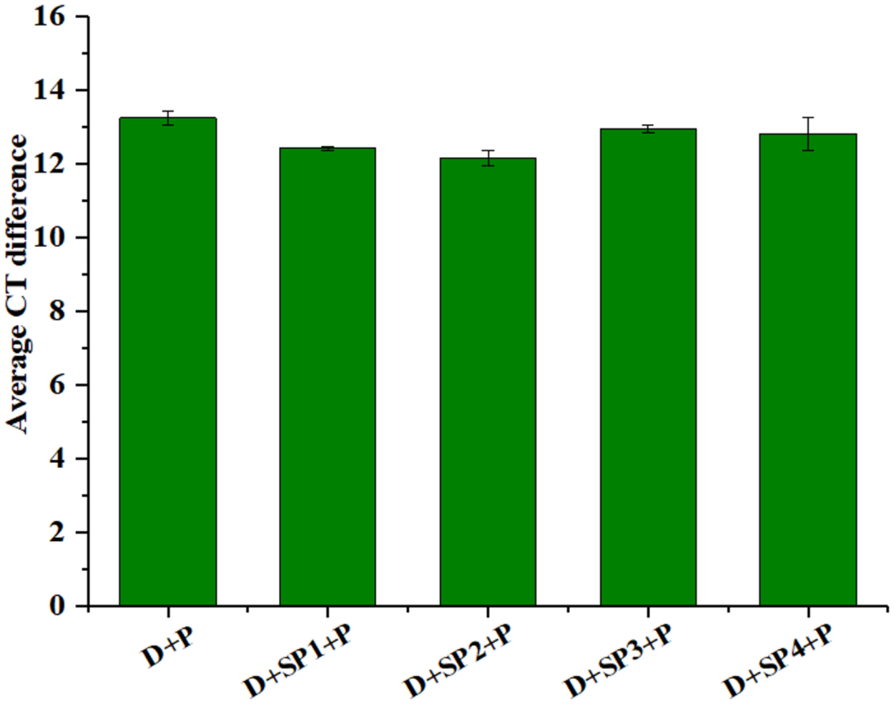

Fig. S2 The novel qPCR inhibition of practical sample. (D: dead cell, P: PMA, SP1: shrimp, SP2: calm, SP3: water collected from shrimp bucket, SP4: water collected from calm bucket)
